# Hot Formability Study of Cr5 Alloy Steel by Integration of FEM and 3D Processing Maps

**DOI:** 10.3390/ma15144801

**Published:** 2022-07-09

**Authors:** Xuewen Chen, Yahui Si, Rongren Bai, Xiaopeng Zhang, Zhipeng Li

**Affiliations:** School of Materials Science and Engineering, Henan University of Science and Technology, 263 Kaiyuan Avenue, Luoyang 471023, China; siyahui@stu.haust.edu.cn (Y.S.); bairongren317@163.com (R.B.); xp99525@163.com (X.Z.); mrlzp597@163.com (Z.L.)

**Keywords:** Cr5 alloy steel, three-dimensional (3D) processing maps, finite element method (FEM), microstructure evolution

## Abstract

Microstructure is an important factor that affects the mechanical properties and service life of forgings. Through the full study of the formability of the material, the internal microstructure of the material can be effectively controlled. In order to accurately describe the formability of materials during thermal processing, 3D hot processing maps containing strains were established in this paper, and the 3D hot processing maps were coupled with the finite element method for simulation calculation. The Cr5 alloy steel was subjected to unidirectional thermal compression at a strain rate of 0.005–5 s^−1^ and temperature range of 900–1200 °C on a Gleeble-1500D thermal simulation machine, in order to obtain the date of true stress and strain. Based on the dynamic material model (DMM), the 3D processing maps of Cr5 alloy steel was established, and the 3D processing maps were associated with the analysis of microstructure evolution during hot deformation. The results show that the optimum thermal deformation conditions are as follows: temperature of 1000–1125 °C, strain rate of 0.01–0.2 s^−1^, and peak power dissipation of 0.41. The 3D processing maps were coupled with the finite element software FORGE^®^ to simulate the hot working process, and the distribution and change of power dissipation and flow instability domain on the metal deformation under different thermal deformation conditions were obtained. The comparison between the simulation results and metallographic images of typical regions of metal deformation shows that they are in good agreement. This method can effectively predict and analyze the formability of materials during hot processing and provide guidance for practical industrial production.

## 1. Introduction

With the development of science and technology, automobiles, ships, bridges, aerospace, and other fields have put forward higher requirements for the required plates and strips; while meeting the mechanical properties, more accurate requirements are put forward for the thickness and shape tolerances of the plates and strips produced by the rolling mill. This requires that the work roll of the mill be able to maintain its contour shape during rolling. As an important stress part of the rolling mill, the backup roller is used to support the work or intermediate rollers, in order to prevent the working roller from deforming during the rolling process, thus affecting the quality of the product [1,2,3]. Large backup rollers are usually forged from large ingots in their entirety. However, due to the high alloying element content and complex composition of steel used for the large backup roller, the composition and microstructure segregation degree of the large ingot is relatively more serious, which increases the difficulty of forging [4]. At the same time, due to the use environment, working state, and other reasons, the backup roller is required to have high fatigue strength, good crack propagation resistance, and excellent wear resistance [5,6]. This requires a full study of the formability of steel forgings for large backup rollers, and a reasonable forging process is aquatinted to homogenize the internal organization of the forging and refine the grains, thereby improving the mechanical properties of the backup roller and prolonging the service life.

The formability of materials depends on the response of the internal microstructure to the temperature, strain rate, and strain during processing. The DMM model-based hot processing map is a characterization of the material’s response to applied processing parameters, in terms of microstructure [7], and it is considered an important means for studying the formability of materials and optimizing the technological parameters of hot processing [8]. The hot processing map can not only be used for making an analysis and prediction on the deformation characteristics and mechanisms of the materials under diverse deformation conditions, but the safe and non-safe areas for hot processing of materials can also be obtained, so as to optimize the process parameters, thus effectively controlling the internal structure of the material and avoiding the occurrence of defects [9].

Based on irreversible thermodynamic theory, in 1987, Prasad and Gegel constructed the DMM model [10], and it is considered to be the link between structural mechanics of plastic deformation continuums with the evolution of microscopic tissues of materials. The power dissipation value, based on the DMM model, was used to describe the relative rate of energy consumed by the microstructure evolution of material during plastic deformation. The power dissipation value represented the strength of the microstructure evolution inside the material. Therefore, the power dissipation graph actually represents the change rate of the internal microstructure of the material during the deformation process of hot working, which is also known as the “microstructure trajectory” [11,12]. However, it does not mean that the higher the power dissipation value is, the better the intrinsic formability of the material is, because the power dissipation value may be higher in the unstable region. Therefore, it is necessary to determine the processing instability zone of the alloy. Prasad et al. [7]. argued that, if the material system does not produce entropy at a rate that at least matches the rate of entropy input through imposed process parameters, the flow of material becomes localized and causes a flow instability. According to the instability criterion, the instability coefficient of material under different processing conditions can be determined. When the instability coefficient is less than 0, it indicates that the material has instability risk under the processing conditions. According to the instability coefficient of the material under different processing conditions, an instability diagram can be constructed to describe the areas that should be avoided during the hot processing of the material. Superposition of the power dissipation and instability maps can build the hot processing map of the material. The optimum deformation zone of material can be determined according to the processing map. In the non-unstable region of the hot processing map, the higher the power dissipation coefficient, the better the formability of the material [13]. Since the DMM model and instability criterion were suggested, the hot processing maps were widely used to study the formability of materials [8,14,15].

However, for many alloys, strain has a greatly affect on microstructure evolution. The traditional 2D hot processing map only represents the power dissipation value, and the instability area distribution in the 2D space, composed of strain rate and temperature under a certain strain, loses sight of the influence of strain on formability; therefore, the formability of materials cannot be comprehensively characterized [16]. Therefore, many scholars began to explore the establishment of a 3D hot processing map, considering the effect of strain on the basis of 2D hot processing map.

Liu J et al., considering strain’s effect on formability, firstly established the 3D hot processing map of AZ31B magnesium alloy-containing strain, obtaining the optimal thermal deformation area of the material. By the combination of the 3D hot processing maps with numerical emulation technology, the changes in the dissipation efficiency and flow instability coefficients of the materials were obtained with the changes of strain, strain rate, and temperature [17]. Yanhui Liu et al., based on the DMM model, established the hot processing map of 1.15C–4.00Cr–3.00V–6.00W–5.00Mo powder high-speed steel and determined the optimal processing range of the material. They found that the strain had a severe impact on the hot processing map; as strain increased, the power dissipation value increased, as well. Additionally, the safety zone had increased [18]. Guo-zheng Quan et al. introduced the activation energy into the hot processing maps, which is based on establishing 3D hot processing maps of Ni80A superalloy, thus determining the processing parameters of DRX mechanism of the metal, under the condition of a lower energy barrier [19]. Based on 3D hot processing maps and the technology for simulation of a finite element, Jian Zeng et al. conducted the process optimization of a flanged cylindrical part of MG-8GD-3Y alloy and carried out thermal reverse extrusion under optimal forming conditions. The results showed that the flanged cylindrical part forming effect was good, surface quality was good, and microstructure and mechanical properties were relatively uniform [20]. Liyan Ye et al. established 3D hot processing maps of 25Cr2Ni4MoV steel, based on the DMM model, and found that strain had a great impact on the hot processing maps. Combined with the microstructure analysis, it was found that the dissipated power coefficient had a good response to the microstructure evolution [21]. Although the 3D hot processing map can effectively consider the strain on the material formability of the study, it also has limitations in application. Whether it is the traditional 2D hot processing map or a 3D hot processing map, it belongs to the static category and can only represent the power dissipation value and instability area at a specific strain, strain rate, and temperature. In the actual hot deformation process, it is necessary to analyze the formability of parts in the whole hot deformation process, in order to ensure that each area of the final part has a good internal organization. Due to the fact that the forging parts are affected by friction, heat exchange, and other factors, as well as the fact that the strain, strain rate, and temperature of different positions inside the forging parts are not the same, static hot processing maps cannot simply and quickly obtain the distribution and change of power dissipation values and instability coefficients of each area of the parts. This undoubtedly increases the difficulty of analyzing the formability of complex parts.

With the rapid development of computer technology and constitutive models, finite element software has been widely used in the field of plastic forming. In recent years, many scholars have established constitutive models to describe the thermal deformation behavior of metals. Sellars [22,23,24,25] established a hyperbolic sinusoidal (Arrhenius flow stress model) material constitutive model that considered the relationship between strain rate and steady-state stress. The model is suitable for materials under various stress states. However, strain softening was not considered in this model. The Johnson–Cook model was proposed by Johnson G.R. and Cook W.H. [26]; it has clear physical meaning and was mainly used to build flow stress model under the conditions of elevated temperature. However, this model is not suitable for the small deformation behavior of materials. Various hardening models have been used to describe the strain hardening behaviour of materials. These include the Voce, Hockett–Sherby, and Asaro. However, these hardening models are mostly used at room temperature and need to be modified when describing the thermal deformation behavior of materials at high temperatures [27,28,29]. Compared with other models, the Hansel–Spittel constitutive model has a wider range of applications. It is widely used to delineate the hot and cold forming behavior of materials in the finite element software, such as FORGE^®^ and QFORM [30,31]. An appropriate constitutive model, combined with the finite element method, can accurately describe the strain field and strain rate field of each part of the metal’s deformation body in the hot processing process. This makes it possible to simulate the formability of materials in the whole hot processing process by coupling the hot processing map with finite element. 

At present, there are few studies that closely combine hot processing maps with the finite element method to realize the dynamic display of power dissipation values and instability areas in various parts of forgings. Additionally, the coupling of 3D hot processing maps and finite element simulation of the forming of Cr5 alloy steel’s large backup roller is still blank. It is one of the bottlenecks of the technology for developing large backup rollers, which can effectively control the microstructure defects, refine the grain, improve the mechanical properties of the large backup roller, and prolong the service life by selecting the best thermal processing parameters. Therefore, it is of great practical significance to analyze the formability and optimize the technological parameters in the whole process of hot processing by using hot processing maps and computer simulation technology.

In this study, the Gleeble-1500D thermal simulation testing machine was used to carry out a thermal compression test to obtain the real stress and strain data of the material. Considering the effect of strain on the thermal deformation behavior of materials, based on the DMM model and hot processing maps theory, 3D hot processing maps, considering the effect of strain, was constructed on the basis of traditional 2D hot processing maps. The effects of strain, strain rate, and temperature on formability were analyzed. The dynamic display of the power dissipation value and distribution and change of instability area on the metal’s deformation body in the process of hot deformation is realized by integrating the 3D hot processing maps with the finite element simulation software FORGE^®^. The formability of different positions inside the forging parts in the process of hot processing was analyzed intuitively, and the simulation results of typical deformation zone were compared with the microstructure image.

## 2. Experimental Materials and Methods

The experimental material was Cr5 alloy steel, and Cr5 alloy steel increased the content of alloying elements, such as Cr, Si, Mo, and V, on the basis of Cr3 alloy steel. The Cr content increased from 3% to 5%, which not only greatly improved the wear resistance and hardening depth of Cr5 alloy steel, but also played a positive role in delaying crack formation and expansion, as well as improving tempering resistance. Cr5 alloy steel is widely used in the manufacture of large backup roller in high-performance large rolling mills [5,6]. In Table 1, chemical composition of Cr5 alloy steel is shown.

**Figure 1:**
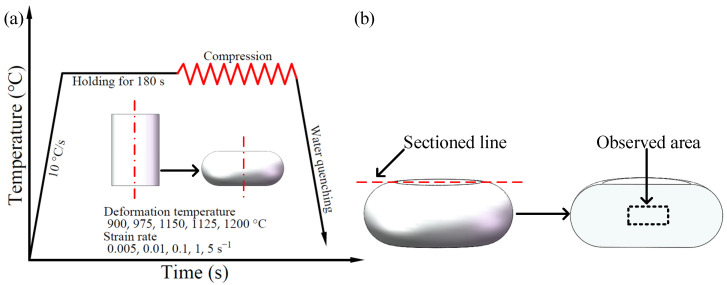
Schematic diagram of experimental process: (**a**) deformation process of hot compression experiment; (**b**) sectioned line and observed area.

Compression specimens were processed into Ø8 × 12 mm cylinders by electrical discharge wire cutting, according to ASTM E209 standard. The Gleeble-1500D thermal mechanical simulator was used for thermal compression testing. The specimen was heated by resistance heating, and the temperature distribution in sample was kept uniformed during heating. The compression test was carried out with constant strain rate, because the Gleeble-1500D thermal mechanical simulator has a function of real-time calculation of true strain during testing, and a strain rate equal to a constant can be achieved. The test was an isothermal test via the thermocouple sensor’s real-time measures of sample temperature and temperature closed-loop controls. When installing the specimen, apply an appropriate amount of graphite lubricant to the indenter and end face of the specimen to decrease the friction between the two. The process of the experiment is shown in Figure 1a. The deformation temperatures of Cr5 alloy steels were 900, 975, 1050, 1125, and 1200 °C, and the strain rates were 0.005, 0.01, 0.1, 1, and 5 s^−1^, respectively. The specimen was first heated at a rate of 10 °C/s to a set deformation temperature and insulated for 180 s to eliminate the temperature gradient and ensure that the material was an internally homogeneous austenitic single-phase structure. Isothermal deformation is then started at the established strain rate, with a maximum deformation of 50%, and the sample was water quenched immediately after the end of deformation to preserve the final microscopic structure. 

As shown in Figure 1b, the hot compressed and water quenched specimens were cut along the axial centerline and mounted, ground, and polished until the surface of the specimen was bright and scratch-free; then, the sample was corroded by saturated picric acid. Finally, the grain structure in the uniform (maximum) deformation zone of the specimen was observed and collected by optical microscope.

## 3. Experiment Results Analysis of Cr5 Alloy Steel 

### 3.1. Influence of Process Parameters on Flow Stress

**Figure 2:**
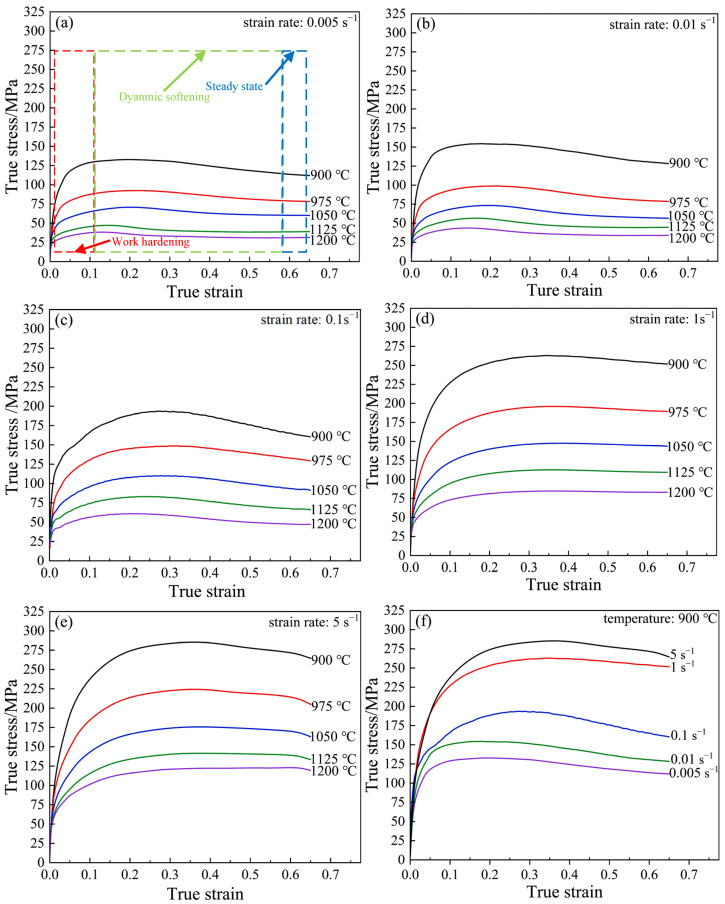
True stress–strain curves of Cr5 alloy steel under various deformation conditions: (**a**) $\dot{\varepsilon }$ = 0.005 s^−1^; (**b**) $\dot{\varepsilon }$ = 0.01 s^−1^; (**c**) $\dot{\varepsilon }$ = 0.1 s^−1^; (**d**) $\dot{\varepsilon }$ = 1 s^−1^; (**e**) $\dot{\varepsilon }$ = 5 s^−1^; (**f**) T = 900 °C.

Figure 2 shows the true stress–strain curves of Cr5 steel under a variety of deformation conditions. It can be seen that the stress had a strong sensitivity to temperature, strain rate, and strain. At the same strain rate, the stress decreased gradually with the increase of temperature. This can be seen in Figure 2a, where the peak stresses were 132.69 and 38.34 MPa at temperatures of 900 and 1200 °C, respectively, and the peak stress was reduced by 94.35 MPa. Peak stress increased as strain rate increased with the same deformation temperature. As shown in Figure 2f, under the deformation conditions of a deformation temperature of 900 °C and strain rates of 0.005 and 5 s^−1^, respectively, the peak stresses were 132.69 and 285.29 MPa, respectively, with an increase of 152.60 MPa. When the strain rate decreased from 1 to 0.1 s^−1^, the stress decreased obviously. The peak stress decreased from 260.71 to 193.58 MPa, with a difference of 67.13 MPa. This is shown in Figure 2. Under most processing conditions, as strain increases, stress changes in roughly three stages, i.e., work hardening (Figure 2a, red wireframe area), dynamic softening (Figure 2a, green wireframe area), and stabilization (Figure 2a, blue wireframe area). As shown in Figure 2b, the true stress–strain curve, which is under conditions of a strain rate and temperature of 0.01 s^−1^ 1050 °C, respectively, at the beginning of the deformation, the stress increases linearly with the increase of the strain. The reason is that, at the beginning of deformation, the dislocation density increases explosively with the increase of deformation, and the migration efficiency seriously affects the diffusion of the dislocation, thus resulting in an increase in deformation resistance and exhibiting process hardening characteristics [32]. As the dislocation density increases, the dynamic recovery softening mechanism is triggered, and the rate of stress growth slows down. As the dislocation density further increases to the critical value of triggering dynamic recrystallization, dynamic recrystallization occurs inside the metal deformation body, and the stress decreases as the dynamic recrystallization softening effect increases. In the later stage of deformation, the softening effect of dynamic recrystallization and recovery mechanisms tends to be balanced with process hardening, and the stress values tends to stabilize and no longer raise with the increase of strain [33].

**Figure 3:**
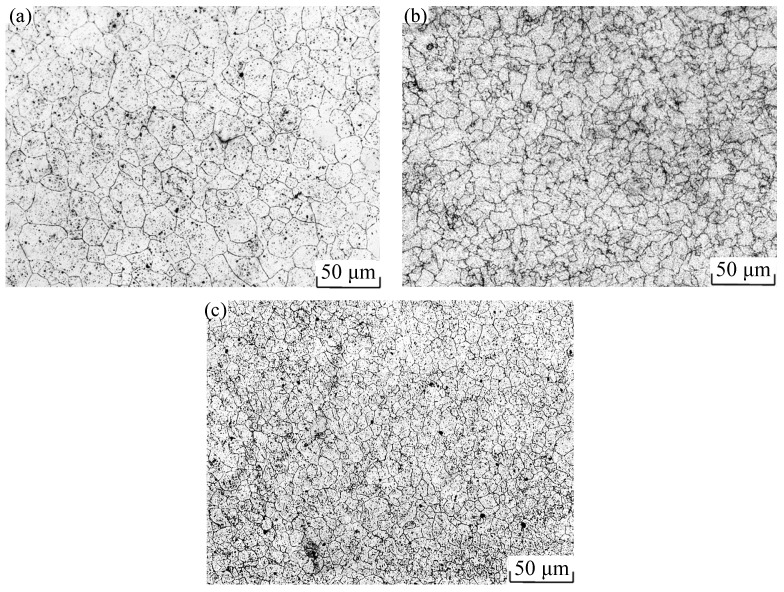
Metallographic photos of Cr5 alloy steel at various deformation conditions: (**a**) T = 1050 °C/ε = 0; (**b**) T = 1050 °C/$\dot{\varepsilon }$ = 0.005 s^−1^/ε = 0.65; (**c**) T = 1050 °C/$\dot{\varepsilon }$ = 0.01 s^−1^/ε = 0.65.

Figure 3 shows the metallographic photos of Cr5 alloy steel under different deformation conditions at 1050 °C. From Figure 3a, it can be seen that, when the deformation temperature of the material is 1050 °C, the grain without deformation is coarse equiaxed grain; the average grain size measured was 27 μm. As shown in Figure 3b, when the strain rate was 0.005 s^−1^ and strain was 0.65, the average grain size measured was 23 μm. As shown in Figure 3b, when the strain rate was 0.01 s^−1^ and strain was 0.65, the average grain size measured was 18 μm. When the strain reached 0.65, the coarse original grain of the material was completely replaced by fine isometric crystals, and there was no obvious immature recrystallized grain at the grain boundary, regardless of the strain rates of 0.005 or 0.01 s^−1^. The results show that the dynamic recrystallization of the Cr5 alloy steel was completed when the temperature was 1050 °C, strain rate was 0.65, and strain rate was 0.005 s^−1^ and 0.01 s^−1^, which coincides with the change trend of the true stress–strain curve under the deformation conditions described above. Therefore, strain has a significant impact on the microstructure evolution and thermal deformation behavior of Cr5 alloy steel. 

### 3.2. Hot Processing Map of Cr5 Alloy Steel

Processing maps, based on DMM models, have been used in a variety of alloys, including alloy steel, zirconium, copper, aluminum, and nickel-based superalloys [34,35,36,37]. In this paper, the processing maps of Cr5 alloy steel were constructed based on the DMM model.

#### 3.2.1. Establishment of Power Dissipation Map

When the temperature and strain are fixed, the change in stress during thermoplastic deformation of an alloy can be described using dynamic material modeling (DMM) [18,38]:

(1) $$\sigma =K{\dot{\varepsilon }}^{m}$$

where σ portrays the stress, $\dot{\varepsilon }$ portrays the strain rate, *K* is a constant, and *m* portrays the strain rate sensitivity coefficient.

At a certain temperature and strain, *m* is constant; that is:

(2) $$m=\frac{\partial \ln\sigma }{\partial \ln\dot{\varepsilon }}$$

The hot deformation process can be considered a closed system. The total power absorbed by a material per unit volume during hot deformation can be expressed by the sum of two complementary functions [10,39]:

(3) $$P=\sigma \dot{\varepsilon }=G+J=\int_{0}^{\dot{\varepsilon }}\sigma d\dot{\varepsilon }+\int_{0}^{\sigma }\dot{\varepsilon }d\sigma$$

In Equation (3), *P* is the total power absorbed per unit volume of the material, *G* is the power dissipation, and *J* is the dissipation allowance. The *G* value represents the power consumption due to plastic deformation, most of which is converted to heat. The *J* value represents the power consumption associated with the change in microstructure during material deformation.

Under the condition of fixed strain and temperature, the rate of change for *J* and *G* constitutes the definition of the strain rate sensitivity coefficient *m*:

(4) $$\frac{dJ}{dG}=\frac{\dot{\varepsilon }d\sigma }{\sigma d\dot{\varepsilon }}=\frac{d\ln\sigma }{d\ln\dot{\varepsilon }}={\left|\frac{\partial (\ln\sigma )}{\partial (\ln\dot{\varepsilon })}\right|}_{\varepsilon ,T}\equiv m$$

Murty et al. [40] thought the changes in *J* represent changes in the microstructure. Since *J* varies nonlinearly, for computational convenience, comparing it with the ideal linear dissipation factor *J*_max_ yields a dimensionless parameter—the power dissipation factor *η*:

(5) $$\eta =\frac{J}{J_{\max}}$$

The material is in an ideal linear dissipation state; that is, *J* reaches the maximum value *J*_max_ when *m* = 1 and *J* = *G* when *m* = 1, so *J*_max_ is:

(6) $$J_{\max}=\frac{\sigma \dot{\varepsilon }}{2}$$

According to Equation (5), the relationship between the power dissipation *η* and strain sensitivity coefficient *m* is as follows:

(7) $$\eta =\frac{2m}{m+1}$$

**Figure 4:**
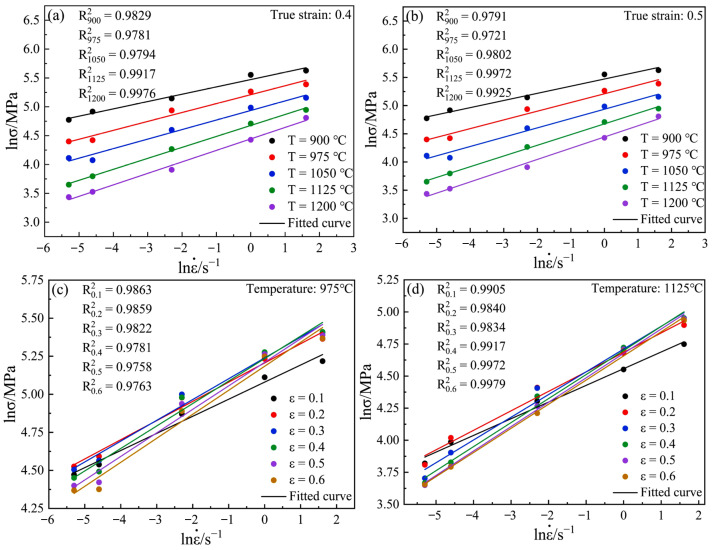
Relationships between $\ln\dot{\varepsilon }$ and lnσ at different temperatures: (**a**) ε = 0.4; (**b**) ε = 0.5; (**c**) T = 975 °C; (**d**) T = 1125 °C.

As shown in Figure 4, according to Equation (2) and the true stress–strain curve of Cr5 alloy steel, the relationship diagram of lnσ and $\ln\dot{\varepsilon }$ can be obtained. Taking true strains of 0.4 and 0.5 and temperatures of 975 and 1125 °C as examples, it can be seen that lnσ and $\ln\dot{\varepsilon }$ have a strong linear relationship under different temperature conditions, and the fitting degrees(R^2^) of all the curves are above 0.97. This shows that Cr5 alloy steel conforms to the assumptions of Equation (2) and is, therefore, able to calculate and construct hot processing maps using all of the above equations. 

At the same strain and deformation temperatures, a and b can be fitted with least squares as cubic polynomials, with the expression of Equation (7):

(8) $$\ln\sigma =a+b\ln\dot{\varepsilon }+c{\left(\ln\dot{\varepsilon }\right)}^{2}+d{\left(\ln\dot{\varepsilon }\right)}^{3}$$

**Figure 5:**
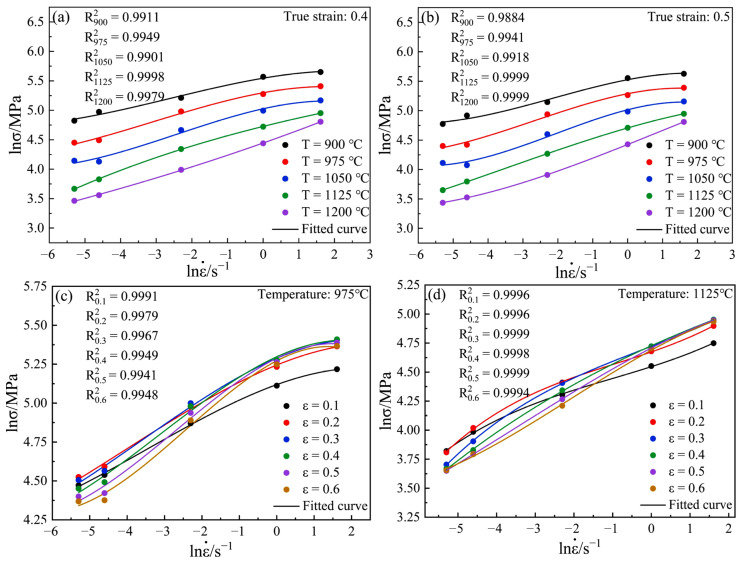
Cubic spline curve fitting of $\ln\dot{\varepsilon }$ and lnσ at different temperatures: (**a**) ε = 0.4; (**b**) ε = 0.5; (**c**) T = 975 °C; (**d**) T = 1125 °C.

As shown in Figure 5, the lnσ and $\ln\dot{\varepsilon }$ of Cr5 alloy steel, under different strain conditions, are fitted with the least squares method to a cubic polynomial. For instance, when the true strains are 0.4 and 0.5, strains 0.4 and 0.5, and temperatures 975 and 1125 °C, the fitting degrees(R^2^) of all the curves are above 0.98. This shows that Equation (7) can be used to describe the relationship between lnσ and $\ln\dot{\varepsilon }$, thus deriving Equation (8):

(9) $$m=\frac{\partial \ln\sigma }{\partial \ln\dot{\varepsilon }}=b+2c\ln\dot{\varepsilon }+3d{\left(\ln\dot{\varepsilon }\right)}^{2}$$

**Figure 6:**
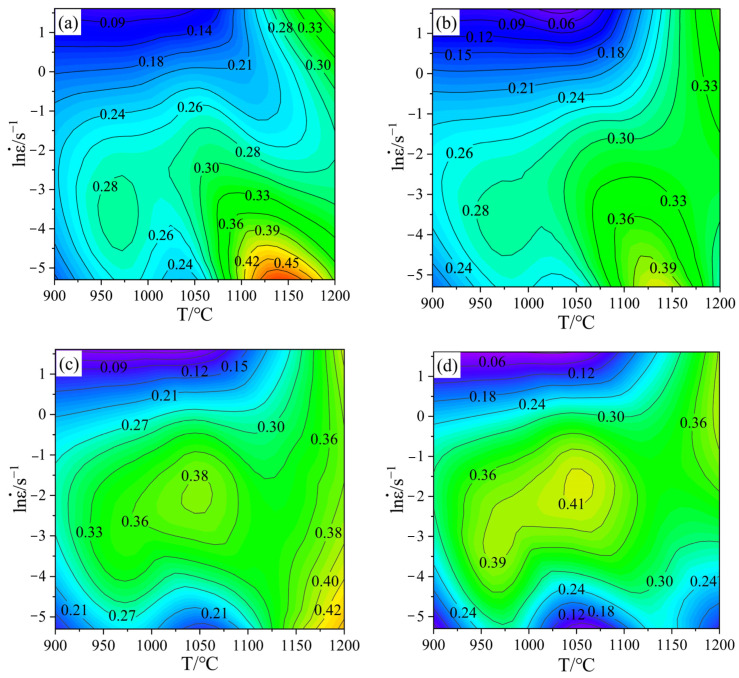
Power dissipation maps at different strains: (**a**) ε = 0.3; (**b**) ε = 0.4; (**c**) ε = 0.5; (**d**) ε = 0.6.

According to Equations (8) and (9), the strain rate sensitivity coefficient m values can be obtained, and the m values can be substituted into Equation (7) to acquire the power dissipation values under the corresponding conditions. Contour maps constructed under the same strain, different strain rates, and temperature conditions are power dissipation maps. As can be seen from the Figure 6, with the increase of strain, power dissipation maps have significant changes, and the peak of the power dissipation value, distribution, and proportion of the high-power dissipation area with a power dissipation value greater than 0.30 are different under different strain conditions, which shows that there is a significant impact, caused by strain, on the dissipation map of Cr5 alloy steel.

#### 3.2.2. Establishment of Instability Map

Ziegler [41] believes that the plastic flow of materials is unstable. If the material system does not produce entropy constitutively at a rate that at least matches the rate of entropy input through imposed process parameters, the flow becomes localized and causes a flow instability. Therefore, he proposes the instability criterion Equation (10) for dynamic material models:

(10) $$\xi (\dot{\varepsilon })=\frac{\partial \ln(\frac{m}{m+1})}{\partial \ln\dot{\varepsilon }}+m<0$$

According to Equations (9) and (10), Equation (11) can be derived:

(11) $$\xi (\dot{\varepsilon })=\frac{m^{\prime }}{m(m+1)}+m=\frac{2c+6d\ln\dot{\varepsilon }}{m(m+1)}+m<0$$

**Figure 7:**
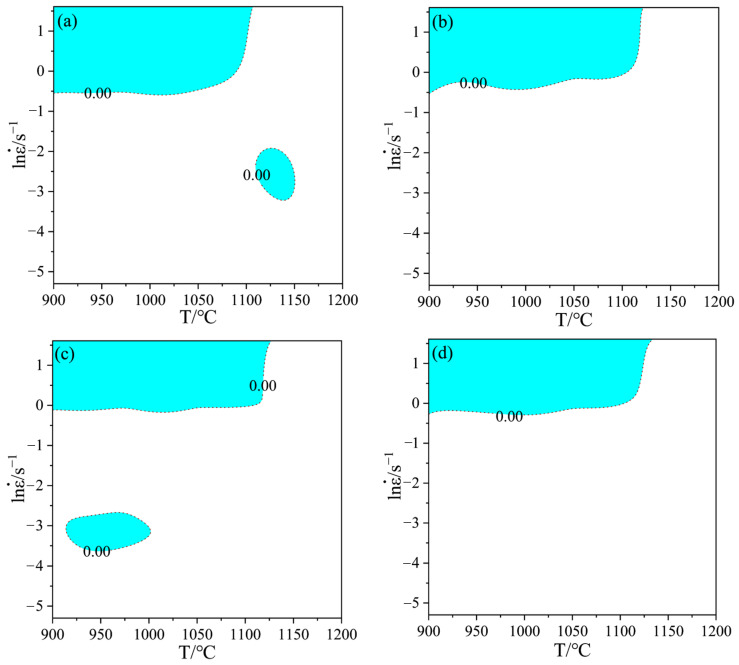
Instability maps at different strains: (**a**) ε = 0.3; (**b**) ε = 0.4; (**c**) ε = 0.5; (**d**) ε = 0.6.

According to Equation (11), the instability map can be obtained under conditions of different strain rates and temperatures at the same strain, and the part less than 0 in Figure 7 (the blue shaded area in the figure) indicates that the region has flow instability. As can be seen from Figure 7, the instability area of Cr5 alloy steel under different strain conditions changes with the increase of strain, but the impact is not significant. The instability area is mainly concentrated in the high strain rate region, with strain rates of 0.6–5 s^−1^ at temperatures of 900–1125 °C.

#### 3.2.3. Establishment of 3D Hot Processing Map

The traditional 2D hot processing map does not consider the influence of strain, but as can be seen from the true stress–strain curve, the material has a significant strain softening effect. Meanwhile, the power dissipation value distribution of the 2D power dissipation maps under different strains of Cr5 alloy steel are analyzed, and it can be found that the strain has a key impact on the processing maps of the material. Accordingly, it is indispensable to establish the processing map the strain effect involved to evaluate the formability of Cr5 alloy steel.

**Figure 8:**
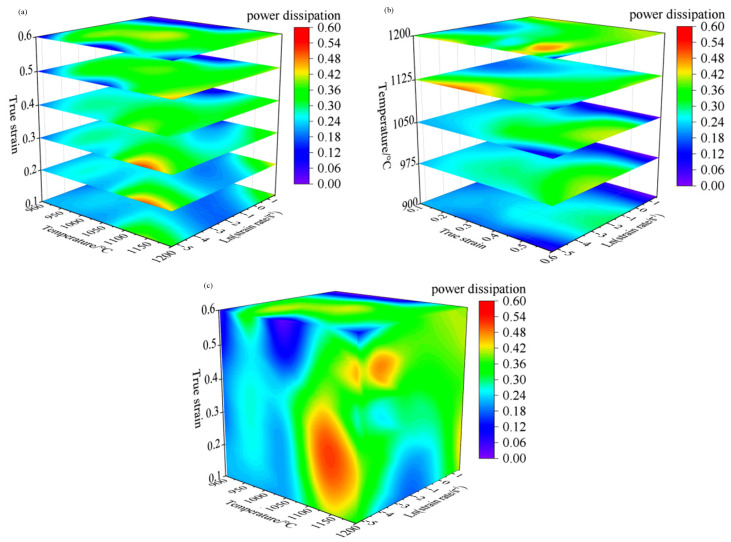
3D power dissipation maps at different hot deformation conditions: (**a**) 3D power dissipation map at strains of 0.1, 0.2, 0.3, 0.4, 0.5, and 0.6; (**b**) 3D power dissipation map at temperatures of 900, 975, 1050, 1125, and 1200 °C; (**c**) 3D power dissipation map.

**Figure 9:**
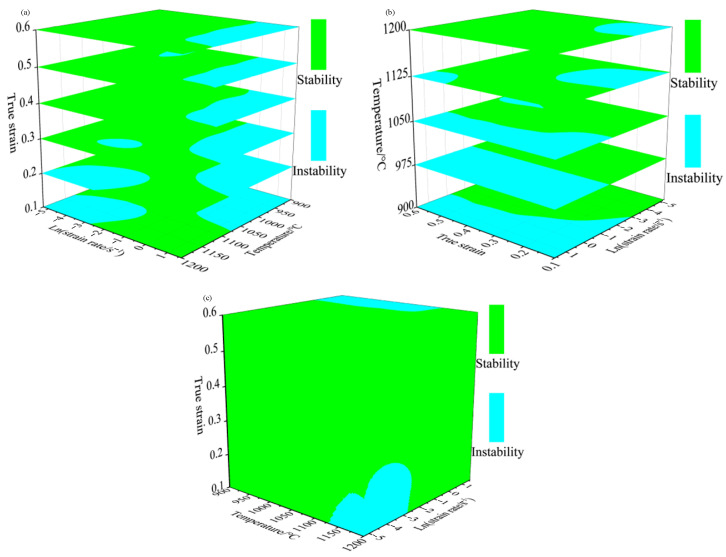
3D instability maps at different hot deformation conditions: (**a**) 3D instability map at strains of 0.1, 0.2, 0.3, 0.4, 0.5, and 0.6; (**b**) 3D instability map at temperatures of 900, 975, 1050, 1125, and 1200 °C; (**c**) 3D instability map.

**Figure 10:**
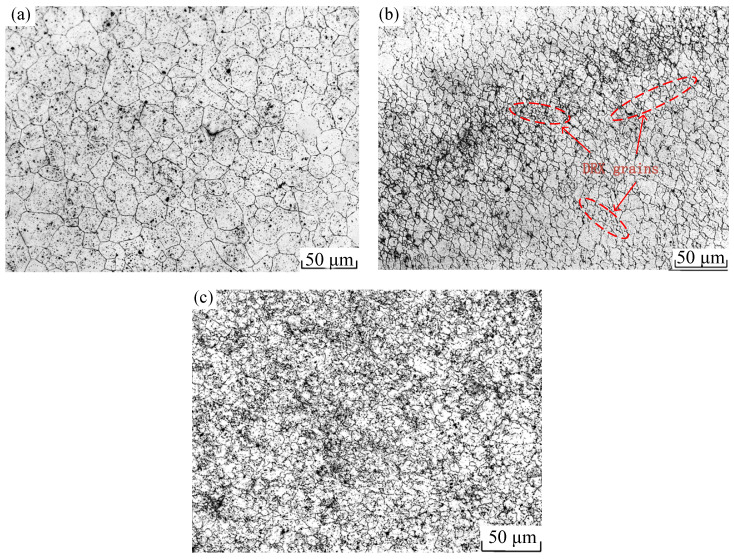
Metallographic photos of Cr5 alloy steel at 1050 °C/0.1 s^−1^: (**a**) ε = 0; (**b**) ε = 0.4; (**c**) ε = 0.6.

Based on the 2D hot processing maps under different strain conditions established above, the temperatures 900–1200 °C, strain rates 0.005–5 s^−1^, and strains 0.1–0.6 range of 3D hot processing maps were established with the strain, temperature, and strain rate as the variation. This is shown in Figure 8 and Figure 9. Figure 8a is a 3D distribution of the power dissipation value at strains of 0.1–0.6, and it can be seen from the figure that, with increase in strain, the proportion of the region with the power dissipation value greater than 0.30 gradually increases, showing that the value is only 6.22% when the strain is 0.1 and 33.98% when the strain is 0.3; the peak value is 61.20% when the strain is 0.5 and 60.67% when the strain is 0.6. It is close to the proportion under strain 0.5. At the same time, with the increase of strain, the power dissipation value under some deformation conditions reaches the peak; for example, when the deformation temperature is 1050 °C, the deformation rate is 0.01–0.5 s^−1^ and when the strain is increased from 0.3 to 0.6, the power dissipation value is gradually increased from 0.27 to 0.41. This is because the change in the power dissipation value is interrelated to the evolution of the microstructure inside the material; with the increase of deformation, the distortion energy of the deformed body augments, thus providing more recrystallization driving force and increasing the probability of forming nuclei, which is conducive to generating more fine recrystallization grains. As shown in Figure 10, the metallographic structure under different strain conditions of temperature 1050 °C and strain rate 0.1 s^−1^ was observed, and it can be seen in Figure 10a,b that the initial grain was relatively coarse. When the strain reached 0.4, dynamic recrystallization occurred inside the material, resulting in more strand-like fine recrystallization grains at the grain boundaries of the original grains. At this time, the dynamic recrystallization was not complete, and the power dissipation value was 0.29. As shown in Figure 10c, when the strain reached 0.6, the fine recrystallization grain at the grain boundary had grown, and the initial grain had been completely replaced by a fine recrystallization grain. At this point, dynamic recrystallization had been completed, and the power dissipation value was 0.40. The main mechanism of microstructure evolution of Cr5 alloy steel under this deformation condition was dynamic recrystallization.

**Figure 11:**
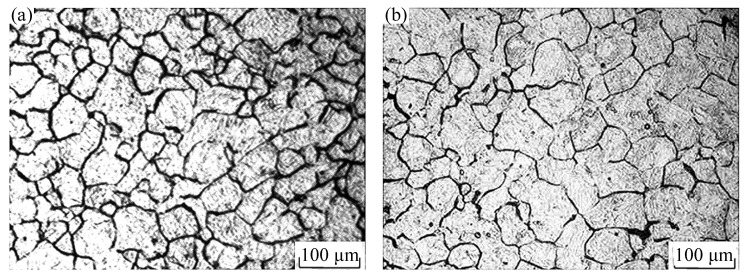
Metallographic photos of Cr5 alloy steel at 0.005 s^−1^/0.6: (**a**) T = 1125 °C; (**b**) T = 1200 °C.

Figure 8b is the 3D distribution of the power dissipation value at temperature values of 900–1200 °C. From this figure, we can see that, with the increase of temperature, the proportion of the area with a power dissipation value greater than 0.30 gradually increased; the proportion was 0.086% when the forming temperature was 900 °C, and it was mainly distributed in the high strain region. The proportion increased to 24.06% when the forming temperature was 1050 °C, and the proportion reached 65.21% when the forming temperature was 1200 °C. This is because the atom activity capacity was enhanced at higher temperatures, thus the resulting dislocation movement was easier to carry out, so that the number of recrystallization-shaped nuclei increased, which is conducive to dynamic recrystallization. However, meanwhile, it can be found that as the deformation temperature rise, the power dissipation value under some deformation conditions decreases. For example, under the conditions that the deformation temperature was 1200 °C, strain was 0.6, and strain rate was 0.005 s^−1^, the power dissipation value reduced from 0.24 at 1125 °C to 0.11. This is because, as the temperature increased, the driving force for recrystallization grain growth also increased significantly to coarse the grain. As shown in Figure 11, i.e., the microstructure of Cr5 alloy steel under strain 0.6, strain rate 0.005 s^−1^, and different deformation temperatures, when the temperature rose from 1125 to 1200 °C, grain coarsening occurred after the recrystallization was completed. The average grain size was 85.93 μm at 1125 °C, and the power dissipation value was 0.24 at this time. When the temperature rose to 1200 °C, the average grain size rose to 102.02 μm, and the power dissipation value reduced to 0.11.

Figure 9 shows the 3D distribution of the instability area under different deformation conditions of Cr5 alloy steel. The green shaded area is the safe area, and the blue shaded area is the instability area. As can be seen in Figure 9a, when the strain was less than 0.3, the instability zone was mainly concentrated in two regions: one was in the temperature range of 1125–1200 °C and strain rate range of 0.005–0.3 s^−1^; the other was in the temperature range of 900–1050 °C and strain rate range of 0.6–5 s^−1^. When the strain was greater than 0.3, the instability region was mainly concentrated in the region with temperature range of 900–1125℃ and strain rate range of 0.6–5 s^−1^. When the strain was larger than 0.3, the impact of strain on the proportion of the instability area was not obvious. The proportion of the instability zone was 21.99% at the strain 0.3, and the proportion of the instability zone was 19.16% when the strain is 0.6; the difference was only 2.83%. With the increase in temperature, the proportion of unstable regions declined gradually, as shown in Figure 9b. The proportion of instability areas was 51.30% at 900 °C, 29.85% at 1050 °C, and 10.72% at 1200 °C.

When selecting the hot processing parameters of the material, the instability areas in the instability maps should be avoided, as these areas are prone to defects, such as flow localization, adiabatic shear bands, micro cracks, and crystal mixing [8,18,37,42]. In principle, it is safe to carry out hot processing in non-instability areas. However, in order to optimize the formability of the material and effectively control the microstructure, the power dissipation value under different processing conditions should also be considered. In the processing maps, the magnitude of the power dissipation values is connected with the mechanism of the microstructure evolution in the region. In the non-instability areas, the microstructure inside the material is better if the power dissipation value is high, thus resulting in better formability [43,44]. Based on the analysis of the hot processing maps and metallographic photos, the optimal hot deformation areas of Cr5 alloy steel are temperature: 1000–1125 °C, strain rate: 0.01–0.2 s^−1^, and strain greater than 0.3.

### 3.3. Hot Formability Analysis of Cr5 Alloy Steel by Coupled 3D Hot Processing Maps and FEM

**Figure 12:**
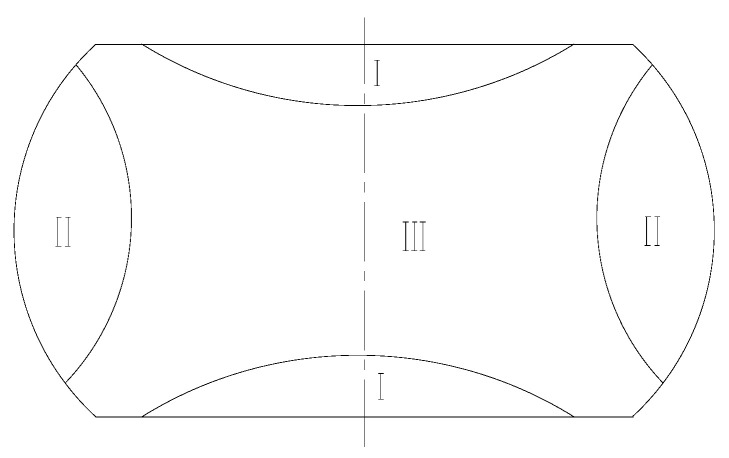
Schematic of the non-uniformity of compressed sample.

While hot processing maps can be applied for determining the optimal processing area of a material, it is a static display, and the actual thermal deformation process is more complex. In the actual processing process, the parts are affected by friction, heat exchange, and other factors, thus resulting in uneven temperature and deformation fields inside the parts. As shown in Figure 12, which is the schematic diagram of the macroscopic deformation of the specimen after thermal compression, according to the degree of deformation, the deformation body is able to be divided to divided into three regions, as follows. Region I. In contact with the upper and lower indenters, it is affected by tangential friction during the deformation process, and it is not easy to produce plastic flow, which is called the difficult deformation area. Region II. The stress state of the region is two pressures and one pull, which is the free deformation area; it is more likely to deform than the region I. Region III is subjected to a three-way compressive stress state, and the deformation resistance is small, which is the large deformation area.

The non-uniformity deformation and temperature fields of a material during hot processing undoubtedly increase the difficulty of the application of the hot processing maps. Integration of hot processing maps with the finite element can realize the dynamic display of power dissipation values and instability areas in various portions of the part during thermal processing, which helps to intuitively, comprehensively, quantitatively, and accurately formulate and optimize the hot processing parameters. 

Through secondary development, the 3D hot processing maps (Figure 8 and Figure 9) of Cr5 alloy steel, established above, are embedded in the finite element software FORGE^®^. The compression of a cylindrical billet is modelled by the FORGE^®^ software. The diameter and initial height of the billet were 8 and 12 mm, respectively. The Hansel–Spittel constitutive model of Cr5 alloy steel was used in the simulation calculation, as established by the author in the previous study [45] and shown in Equation (12). Other simulation parameters are shown in Table 2.

(12) $$\begin{array}{ll} \sigma = & 9.135695\cdot 10^{15}\cdot e^{0.001413T}\cdot \varepsilon^{0.174840}\cdot {\dot{\varepsilon }}^{-0.075205}\cdot e^{\frac{-0.005456}{\varepsilon }}\\ & \cdot {\left(1+\varepsilon \right)}^{-0.001042T}\cdot e^{0.030937\varepsilon }\cdot {\dot{\varepsilon }}^{0.000212}\cdot T^{-4.713008} \end{array}$$

According to the same deformation temperature and rate as the experiment, the thermal compression process of Cr5 alloy steel under different deformation conditions was simulated and calculated. The dynamic distribution of power dissipation values and instability areas of Ø8 × 12 mm cylindrical specimens under different deformation conditions were obtained. Typical simulation conditions and parameters were chosen, in order to avoid repeating the same conclusions.

**Figure 13:**
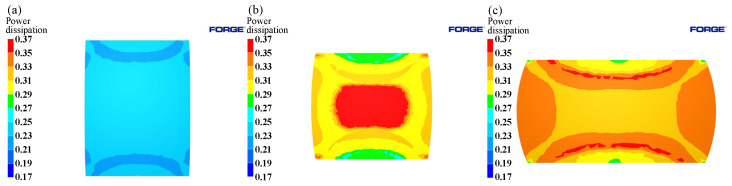
Distributions of power dissipation at 1125 °C/0.1 s^−1^: (**a**) 10%; (**b**) 30%; (**c**) 50%.

The distribution of power dissipation values with a deformation condition of 1125 °C/0.1 s^−1^ was shown in Figure 13. According to the figure, it is evident that, with the increase of the compression deformation, the area with a power dissipation value greater than 0.31 increased rapidly, which was mainly concentrated in the large deformation (Region III) and free deformation (Region II) regions. The power dissipation value in the difficult deformation region (Region I) was relatively low, but it also increased with the increase of compression deformation.

**Figure 14:**
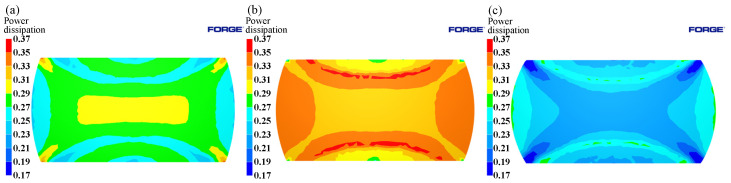
Distributions of power dissipation at 1125 °C/50%: (**a**) 0.01 s^−1^; (**b**) 0.1 s^−1^; (**c**) 1 s^−1^.

**Figure 15:**
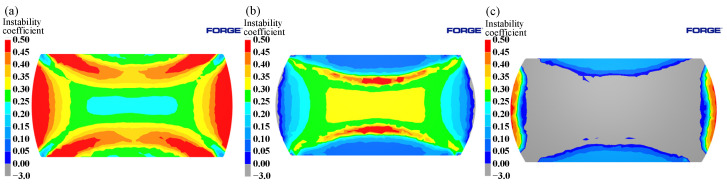
Evolution of flow instability at 1125 °C/50%: (**a**) 0.01 s^−1^; (**b**) 0.1 s^−1^; (**c**) 1 s^−1^.

The distribution of power dissipation values at a temperature of 1125 °C, press quantity of 50%, and different strain rates are shown in Figure 14. As the strain rate decreases, the power dissipation value increases rapidly. Peaks are reached at strain rates of 0.1 s^−1^, followed by a decrease at strain rates 0.01 s^−1^. This is due to the fact that dynamic recrystallization takes more time at lower strain rates; however, at higher deformation temperatures, the grains will grow further after completion, resulting in a decrease in power dissipation values. As shown in Figure 15, as the strain rate increased, an instability area (shade of gray) appeared at the edge of the cylinder at a strain rate of 0.1 s^−1^. As the strain rate increased, the instability area shifted to the center of the cylinder and expanded; the results coincide with the results of the hot processing maps analyses.

**Figure 16:**
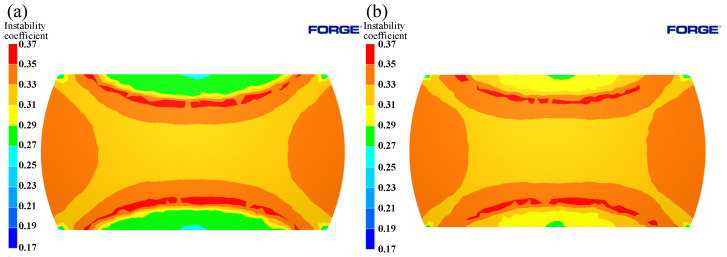
Distributions of power dissipation at 0.1 s^−1^/50%: (**a**) 1050 °C; (**b**) 1125 °C.

**Figure 17:**
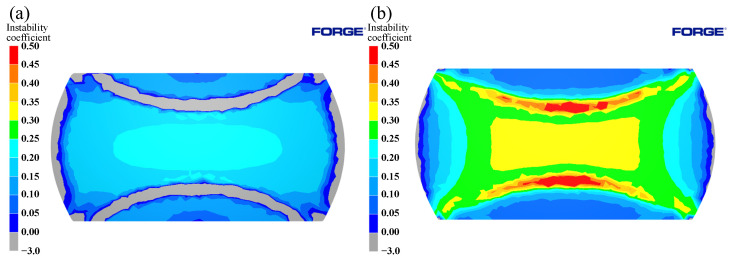
Evolution of flow instability at 0.1 s^−1^/50%: (**a**) 1050 °C; (**b**) 1125 °C.

The effects caused by different deformation temperatures on the power dissipation and flow instability distribution are shown in Figure 16 and Figure 17. From Figure 16, it can be seen that the high-power dissipation value area increased with increasing temperature, and Region I varied significantly. As shown in Figure 17a, instabilities occur at the edge of the cylinder and junction of the three typical regions under a temperature of 1050 °C. Due to the uneven deformation of the I, II, and III Regions, the shear strain at the junction is highly concentrated, and adiabatic shear belts appeared, thus leading to the emergence of instability areas. As shown in Figure 17b, with the increase of temperature, the area of instability area decreased. At 1125 °C, instability occurred only at the edge of cylinder, which was consistent with the distribution of instability area in the hot processing maps.

**Figure 18:**
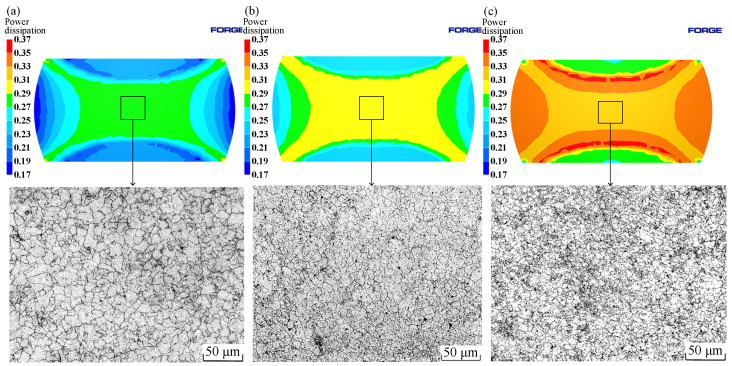
Distributions of power dissipation at 1050 °C/ 50%: (**a**) 0.005 s^−1^; (**b**) 0.01 s^−1^; (**c**) 0.1 s^−1^.

The simulation calculation results of the power dissipation values at different strain rates under the deformation condition of 1050 °C/50% were compared with the metallographic photos, as shown in Figure 18. In the range of 0.005–0.1 s^−1^, with an increase in strain rate, the power dissipation increased gradually. When the strain rate was 0.005 s^−1^, the power dissipation of the sample core was 0.28. When the strain rate was 0.01 s^−1^, the power dissipation value of the sample core was 0.30. When the strain rate was 0.1 s^−1^, the power dissipation value of the sample core was 0.32. It can also be found that the grain size decreased gradually with the increase of strain rate, and the average grains were 23, 18, and 13 μm, respectively. Higher power dissipation has finer grain structure. The experimental results were consistent with the simulation results.

**Figure 19:**
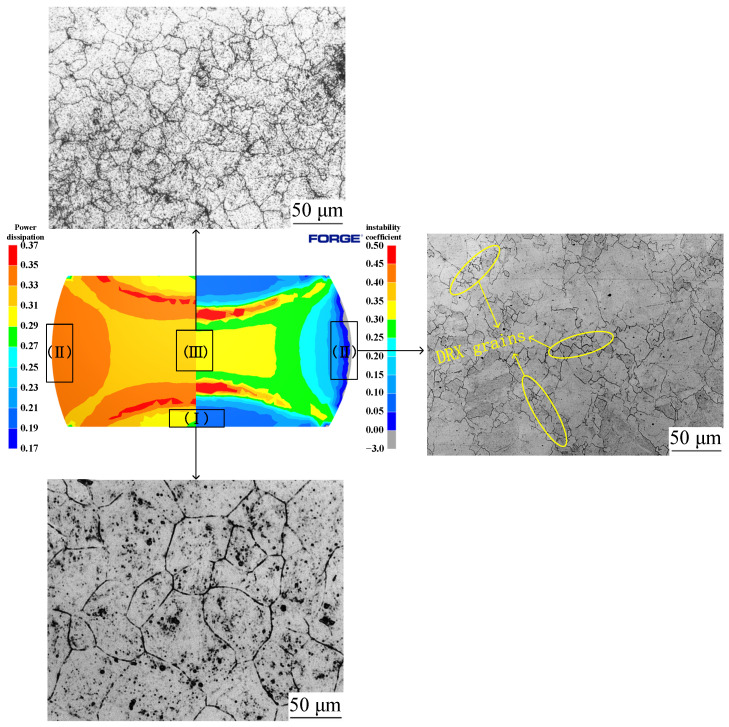
Metallographic photos, power dissipation, and instability coefficient at 1125 °C/0.1 s^−1^/50%: (I) Region I, (II) Region II, (III) Region III.

The simulation results of 1125 °C/50%/0.1 s^−1^ with deformation conditions were compared with metallographic photographs, as shown in Figure 19. It can be seen from the figure that, although there was no instability in Region I, it had the lowest power dissipation value (0.28) in the three regions, and the grains in this region were also the coarsest; there was no obvious dynamic recrystallization behavior, and the microstructure was not ideal. Region III had the largest amount of deformation, which caused dynamic recrystallization to easily occur. From the figure, it can be seen that the power dissipation value of this region was 0.32 and the grain was the smallest; the coarse original grain was replaced by a fine recrystallization grain, which means it had completed dynamic recrystallization, and microscopic organization was more ideal. As can be seen from Figure 13, when the press quantity increased from 30% to 50%, Power dissipation of Region III decreased from 0.36 to 0.32. This is due to the grain grew after dynamic recrystallization, thus resulting in a slight decrease in the dissipation value. Region II had the highest power dissipation value (0.34) in three zones, as well as an instability zone. As can be seen from the metallographic photographs, there were many tiny dynamic recrystallization grains at the initial grain boundaries in this region, and vigorous dynamic recrystallization was underway. It can also be seen that, in addition to the fine recrystallization grains in the microscopic tissues of this region, there were also coarse crystals; coarse grains can easily lead to instability in this region. A good agreement exists between the simulation results and microstructure photographs, which show that the way of integrating the 3D processing maps into FORGE^®^ can effectively predict and analyze the microstructure evolution and formability of Cr5 alloy steel at different times, as well as in different positions, during thermal processing, and provide guidance for the simulation calculation and actual production of a subsequent large backup roller.

## 4. Conclusions

In this paper, the traditional 2D and 3D hot processing maps of Cr5 alloy steel were obtained, based on thermal compression test data, and the 3D processing map was embedded in FORGE^®^ for simulation calculation through program’s secondary development. Combined with metallographic photo analysis, the formability of Cr5 alloy steel during thermal processing was studied.

(1) Changes in the true stress of Cr5 alloy steel were strongly connected to the thermal deformation parameters. The true stress increased with the increase of strain rate and reduction of deformation temperature. At the early stage of deformation, the stress increased swiftly, reached the peak value, and then decreased. As the deformation proceeded, the stress value became stable, thus showing typical dynamic recrystallization characteristics.
(2) At high temperatures, the effect of strain on the formability of Cr5 alloy steel can be characterized by 3D hot processing maps. As the temperature and strain increased, the percentage of high-power dissipation areas increased. As the temperature and strain increased, the percentage of high-power dissipation areas increased, mainly in the following parameters: temperature, 1000–1125 °C; strain rate, 0.01–0.2 s^−1^, the peak power dissipation in these areas is 0.41. With increasing temperature, the area of instability decreased. The impact of strain on the instability area was not significant, which was mainly concentrated in two regions: one is in the temperature range of 1125–1200 °C and strain rate range of 0.005–0.3 s^−1^; the other is in the temperature range of 900–1050 °C and strain rate range of 0.6–5 s^−1^. Combined with metallographic analysis, the optimal hot processing area of Cr5 alloy steel was: temperature, 1000–1125 °C; strain rate, 0.01–0.2 s^−1^; strain > 0.3.
(3) By integrating hot processing maps with finite element, the dynamic display of power dissipation and the instability region of the metal deformation body under different deformation conditions can be obtained. The variation trend of the power dissipation and instability regions with temperature, strain rate, and strain were calculated by simulation and are in good agreement with the 3D thermal processing chart.
(4) Through the simulation of the compression process of Cr5 alloy steel sample, it was found that the power dissipation value and instability coefficient of each part of the sample were not the same. There were obvious differences that should be taken into account when optimizing the hot processing parameters of the material. It is not that the area with higher dissipation value was smaller in grain size, so it was necessary to analyze and discuss the whole change process of the dissipation value. The simulation results of the typical deformation areas were in good agreement with the metallographic photos. This method can effectively analyze the formability of different parts of the material in the process of hot processing and provide guidance for the simulation calculation of formability and optimization of actual production process parameters of the stress support roll for large Cr5 alloy.

## Figures and Tables

**Table 1:** Chemical compositions of Cr5 alloy steel (mass percentage: wt.%).

Fe	C	Mn	Cr	Ni	Mo	V
margin	0.51	0.46	4.94	0.44	0.53	0.16

**Table 2:** Process parameters used in simulation.

Heat Exchange Coefficient of Material/Die Interface (W/m^2^/K)	Coulomb Friction Coefficient of the Material/Die Interface	Die Temperature (°C)	Type of Mash	Size of Mesh (mm)
1000	0.1	250	tetrahedron	0.2

## Data Availability

The data presented in this study are available on request from the corresponding author. The data are not publicly available due to these data are part of ongoing research.
